# Audit of use of stiripentol in adults with Dravet syndrome

**DOI:** 10.1111/ane.12611

**Published:** 2016-05-27

**Authors:** S. Balestrini, S. M. Sisodiya

**Affiliations:** ^1^ Department of Clinical and Experimental EpilepsyNIHR University College London Hospitals Biomedical Research CentreUCL Institute of NeurologyLondonUK; ^2^ Epilepsy SocietyChalfont‐St‐PeterBuckinghamshireUK; ^3^ Neuroscience DepartmentPolytechnic University of MarcheAnconaItaly

**Keywords:** antiepileptic drugs, epilepsy, seizures, stiripentol

## Abstract

**Objectives:**

There are very few data available in the literature on the use of stiripentol in adults with Dravet syndrome (DS). DS cases are increasingly recognized in adulthood, and more children with DS now survive to adulthood. The aim of the study was to document the effectiveness and tolerability of stiripentol in adults with DS.

**Material and methods:**

We conducted an observational clinical audit in the epilepsy service of the National Hospital for Neurology and Neurosurgery, London (UK).

**Results:**

We included 13 adult subjects with DS (eight females, five males). The responder (defined as more than 50% reduction in all seizure types) rate was 3/13 (23%) at 36 months. The following other outcomes were reported: seizure exacerbation (3/13, 23%), no change (3/13, 23%), less than 50% reduction in seizures (2/13, 15%), more than 50% reduction in generalized tonic‐clonic seizures but no other seizure types (1/13, 8%), undefined response (1/13, 8%). The retention rate was 62% after 1 year and 31% after 5 years. Adverse effects were reported in 7/13 (54%): the most frequent were anorexia, weight loss, unsteadiness and tiredness. Withdrawal due to adverse effects occurred in 3/13 (23%).

**Conclusions:**

Compared with previous studies on children with DS, our results show a lower responder rate and a similar tolerability profile. Stiripentol can be effective with a good tolerability profile. Our audit is small, but supports the use of stiripentol in adults with DS when first‐line treatments are ineffective or not tolerated, in keeping with published guidelines.

## 1 Introduction

Dravet syndrome (DS) is a severe genetic epileptic encephalopathy with onset in infancy, often associated with drug‐resistant epilepsy, developmental slowing, cognitive impairment, occurrence of status epilepticus and elevated risk of early mortality.^1^, ^2^, ^3^ In most cases, the cause is a mutation in the voltage‐gated sodium channel type I alpha subunit gene, *SCN1A*.^4^ The main challenges include seizure control, prevention of status epilepticus and optimizing development of cognitive function, where possible. The adult phenotype has been described in a number of previous studies.^2^, ^5^, ^6^, ^7^, ^8^ Adjustment of treatment may be associated with improved seizure control, cognition and quality of life, even into later adult life.^2^, ^6^ Sodium channel blockers, for example, carbamazepine, oxcarbazepine and phenytoin, should usually be avoided as they can aggravate both seizures and interictal EEG.^9^ Lamotrigine can also cause seizure exacerbation,^10^ although has been reported to be beneficial in some cases.^11^ Effective treatment strategies include valproic acid, clobazam, topiramate, levetiracetam, fenfluramine and bromides,^12^, ^13^, ^14^ or dietary therapies (ketogenic or modified Atkins diet).^15^ There are ongoing studies on the use of cannabidiol in children with DS.^16^ Stiripentol was licensed under the European Medicine Agency Orphan Drug scheme in 2001 and is the only treatment for which a randomized, placebo‐controlled trial has been performed in children with DS,^17^ showing that significantly more children with DS on stiripentol adjunctive therapy were seizure‐free, or experienced at least a 50% reduction in seizure frequency, compared with placebo. Very few data are available about the use of stiripentol in adults, with reports for fewer than fifteen DS cases older than 18 years,^18^, ^19^, ^20^, ^21^ and twelve healthy adult volunteers.^22^ We conducted a clinical audit to document the effectiveness and tolerability of stiripentol in adults with DS.

## 2 Material and Methods

This project was reviewed and registered as an observational clinical audit at the National Hospital for Neurology and Neurosurgery, London (UK). Ethics committee approval is not required for an audit, as clinical audit by definition does not involve intervention beyond usual clinical management.^23^

We included all adults (>18 years) with DS attending the epilepsy service with previous or current treatment with stiripentol, from January 2001 to July 2015. DS was defined according to the following clinical criteria: seizure onset in the first year of life, with prior normal psychomotor development, intractable epileptic seizures triggered by infections and increased temperature, with no evidence of structural‐metabolic aetiology.^1^ Cases were identified by reviewing all known DS cases from clinical registers, reviewing all requests for *SCN1A* mutation testing by the hospital clinical genetics service and local hospital pharmacy prescriptions of stiripentol. Demographic and clinical information were gathered.

The following variables were included in the analysis: age, gender, age at clinical diagnosis, age at genetic testing, *SCN1A* mutation details, seizure types, history of status epilepticus, antiepileptic drugs (AEDs) tried before the introduction of stiripentol, age at introduction of stiripentol, interval between genetic diagnosis and introduction of stiripentol, AEDs initially used with stiripentol, mean monthly seizure frequency before the introduction of stiripentol and while on stiripentol treatment, maximum dose of stiripentol, adverse effects during treatment with stiripentol, and total duration of exposure to stiripentol. Seizure exacerbation was defined as an increase of more than 25% in seizure frequency compared with seizure frequency before the introduction of stiripentol.

Statistical analysis was performed using Stata/IC V.11.1 (Stata, TX, USA). Descriptive statistics (mean, minimum, maximum, standard deviation, and median—when appropriate) were used.

## 3 Results

We identified 32 adult cases with DS in our service. Thirteen adults with DS and previous or current treatment with stiripentol were identified and comprised eight females and five males. DS was diagnosed on the basis of clinical findings, at a mean age of 24 (SD 11, range 2–41). Genetic analysis was undertaken at a mean age of 27 (SD 9, range 12–43). *SCN1A* mutation or rearrangements were found in 12 of the 13 patients included. All cases had multiple seizure types. Eight patients (62%) had a history of status epilepticus prior to the introduction of stiripentol. In five cases (29%), stiripentol was introduced in adolescence and continued into adulthood. The mean age at introduction of stiripentol was 26 years (SD 12, range 12–47). In four subjects, it was introduced seven to ten years (median 7.5) before the genetic analysis. In the other nine cases, it was introduced a few months to 6 years (median 1 year), after positive genetic testing for *SCN1A* mutation. At the time of stiripentol introduction, the mean number of AEDs already tried was 9 (range 3–15), and it was used with one to four concurrent AEDs. None of the cases was ever on monotherapy with stiripentol. Details of *SCN1A* mutations, seizure types, AED history and age at introduction of stiripentol are shown in Table 1.

**Table 1:** Details of *SCN1A* mutations, seizure types, AED history, and age at introduction of stiripentol

Case ID	*SCN1A* mutations	Type of mutations	Seizure types^a^	AEDs tried before the introduction of stiripentol^b^	Age at introduction of stiripentol (years)
1	c.4568T>C	Missense	Atonic, GTC, myoclonic	Carbamazepine, clonazepam, ethosuximide, lamotrigine, levetiracetam, nitrazepam, phenobarbitone, phenytoin, piracetam, primidone, topiramate, vigabatrin	41
2	c.80G > C; c.3749C > T; c.3706‐2A > G	Missense, missense, splice‐site	Focal, GTC, tonic	Carbamazepine, clonazepam, gabapentin, lamotrigine, levetiracetam, phenobarbitone, phenytoin, sodium valproate	15
3	c.2792G>A	Missense	Focal, GTC	Clobazam, lacosamide, lamotrigine, levetiracetam, pregabalin, topiramate, zonisamide	44
4	c.4369_4372dupCTGT	Premature STOP codon	Focal, GTC, myoclonic	Carbamazepine, clobazam, ethosuximide, oxcarbazepine, piracetam	29
5	c.603‐2A>G	Splice‐site	Focal, sGCT	Carbamazepine, ethosuximide, lamotrigine, levetiracetam, phenobarbitone, phenytoin, pregabalin, topiramate, vigabatrin	25
6	c.429delGT	Premature STOP codon	Focal, GTC, myoclonic	Carbamazepine, clonazepam, ethosuximide, lamotrigine, piracetam	14
7	c.2244G>A	Premature STOP codon	Focal, GTC, myoclonic	Acetazolamide, carbamazepine, clobazam, clonazepam, diazepam, ethosuximide, lamotrigine, levetiracetam topiramate, vigabatrin	16
8	c.4913T>C	Missense	Focal, GTC, myoclonic	Carbamazepine, gabapentin, oxcarbazepine, phenytoin, sodium valproate, topiramate, vigabatrin	23
9	c.5639G>A	Missense	Atypical absences, focal, myoclonic	Primidone, topiramate	47
10	c.2837G>A	Missense	GTC, myoclonic	Clobazam, lamotrigine, levetiracetam, phenytoin	28
11	c.2293G>A	Missense	Atypical absences, focal, GTC	Acetazolamide, carbamazepine, clonazepam, lamotrigine, nitrazepam, topiramate, vigabatrin, zonisamide	30
12	Not detected	–	Absences, atonic, clonic, focal, GTC, myoclonic, tonic	Carbamazepine, gabapentin, lamotrigine, phenytoin, vigabatrin	12
13	c.5359G>A	Missense	Atonic, clonic, focal, GTC, hemi‐clonic, myoclonic, tonic	Carbamazepine, clobazam, clonazepam, gabapentin, lamotrigine, phenobarbitone, phenytoin, topiramate	12

AED, antiepileptic drugs; GTC, generalized tonic clonic seizures; sGTC, generalized tonic clonic seizure with apparent focal onset.

a Seizure types were clinically determined.

b AEDs are not listed in the temporal order of introduction.

### 3.1 Effectiveness

The frequency of seizures (of any type) at the time of stiripentol introduction varied from daily to weekly. The daily dose varied from 250 mg to 3000 mg with a mean maximum daily dose of 1604 mg. Over an average time of exposure to stiripentol of 42 months (range 3–139), more than 50% reduction in frequency of all seizure types was reported in three cases (23%). The other outcomes were: less than 50% reduction in frequency of all seizure types in two cases (15%), no change in seizure frequency in three cases (23%), more than 50% reduction in frequency of generalized tonic‐clonic seizures (GTCs) but no change in frequency of other seizure types in one case (8%), seizure exacerbation in three cases (23%) and undefined response in one case (8%). One case had initially more than 50% reduction in frequency of GTCs, from weekly to monthly GTCs with a 45 day period without any GTCs, and became then free of GTCs for about 12 months, followed by GTCs recurrence precipitated by intense physical exercise. Before introduction of stiripentol, eight patients (62%) had had from one to multiple episodes of status epilepticus. Of these cases, four had no further episodes of status after the introduction of stiripentol until the latest follow‐up, over an average time of 43 months (range 3–139) on stiripentol.

Withdrawal due to lack of efficacy occurred in four cases (31%). In seven cases (54%), treatment was still ongoing at the time of the audit (July 2015), after they had been on stiripentol for an average time of 62 months (range 10–139). The following medication changes were undertaken in these seven cases during the course of treatment with stiripentol: in two clobazam was introduced; in one carbamazepine was withdrawn and clobazam introduced; in one phenytoin was withdrawn; in one sodium valproate was introduced; in one topiramate was withdrawn, sodium valproate, levetiracetam, zonisamide and perampanel were introduced sequentially (zonisamide was soon withdrawn due to lack of effectiveness) and a vagus nerve stimulator was recently inserted; one did not have any changes to concurrent AEDs. Five cases (39%) were on clobazam and 10 cases (77%) were on sodium valproate, as concurrent treatment when stiripentol was initiated. In three cases, (23%) clobazam and in one case (8%) sodium valproate were introduced later on during the course of treatment with stiripentol. Overall, at some point eight cases (62%) had concurrent treatment with clobazam and 11 cases (85%) with sodium valproate. A summary of stiripentol effectiveness in the audited cohort is reported in Table 2.

**Table 2:** Summary of stiripentol effectiveness and tolerability

Case ID	Concurrent AEDs initially associated with stiripentol	Seizure frequency before the introduction of stiripentol	Maximum dose of stiripentol (mg)	Concomitant AEDs modification with introduction of stiripentol	Seizure frequency while on stiripentol	More than 50% reduction in frequency of GTCs	Reduction of episodes of status epilepticus	Adverse effects	Withdrawal (reason)	Time of exposure to stiripentol until withdrawal^a^ or last follow‐up (months)
1	Clobazam, sodium valproate, zonisamide	4–17 GTCs per month	750	No	Exacerbation of seizures (GTCs on a daily basis, increase of myoclonic seizures) and several episodes of status	No	No	None	Yes (lack of efficacy)	3^a^
2	Clobazam, topiramate	Daily	1750	na	Daily	No	na	None	Yes (lack of efficacy)	72^a^
3	Carbamazepine, sodium valproate	3–4 GTCs monthly, 2–3 focal seizures daily	2000	Reduction of sodium valproate (carbamazepine was withdrawn 5 months after introduction of stiripentol)	1–2 GTCs monthly, 5–8 focal szs monthly	Yes	na	Unsteadiness and sedation (1000 mg daily), lethargy (1500 mg), loss of appetite and weight (1750)	Ongoing	48
4	Levetiracetam, sodium valproate	3 GTCs monthly, myoclonic seizures daily	na	Withdrawal of oxcarbazepine	Unchanged	no	na	Anorexia and weight loss	Yes (lack of efficacy and adverse effects)	6^a^
5	Sodium valproate, zonisamide	Weekly, clusters every few weeks	1500	Reduction of zonisamide and sodium valproate	No clusters, reduction in hospitalisations/emergency room visits	Yes	Yes	Weight loss	Ongoing	10
6	Clobazam, sodium valproate	Weekly	3000	na	Initially 1–2 GTCs per month and 45 days free of GTCs, more recently free of GTCs for about 12 months	Yes (seizure‐free)	na	Nausea, unsteadiness, abdominal pain, myelodysplasia (thrombocytopenia and neutropenia)	Ongoing, with dose adjustments, haematological review and regular monitoring	80
7	Sodium valproate	5–19 seizures per month	750	na	9–33 seizures per month	No	No	None	Yes (lack of efficacy)	31^a^
8	Lamotrigine, levetiracetam	Weekly	2000	Withdrawal of carbamazepine	Weekly	No	Yes	None	Ongoing	34
9	Sodium valproate	Focal seizures daily	1000	Reduction of sodium valproate	Focal seizures daily	No	na	Increased aggression and diarrhoea	Yes (adverse effects)	3^a^
10	Sodium valproate, topiramate	4 seizures per month	750	No	11 seizures per month	No	No	Loss of appetite, unsteadiness and increased tremor	Yes (adverse effects)	4^a^
11	Clobazam, levetiracetam, phenytoin, sodium valproate	Daily	2250	Reduction of clobazam (phenytoin was withdrawn 8 months after introduction of stiripentol)	Reduction of seizure frequency, mainly of clusters of GTCs	No	Yes	Loss of appetite and weight, tiredness (when on maximum dose)	Ongoing	42
12	Clobazam, topiramate	Daily	2500	Withdrawal of topiramate and introduction of sodium valproate	Focal seizures daily, GTCs monthly	Yes	Yes	None	Ongoing	139
13	Levetiracetam, sodium valproate	Weekly	1000	Reduction of sodium valproate	Initial slight reduction of seizure frequency	No	Yes	None	Ongoing	79

na, Data not available; GTCs, generalized tonic‐clonic seizures; a, cases for which stiripentol was withdrawn.

### 3.2 Tolerability

Adverse effects were not systemically sought as this was not a prospective study, but were reported in seven cases (54%). These were: anorexia (n=4), weight loss (n=4), unsteadiness (n=3), tiredness/somnolence (n=2), nausea (n=1), abdominal pain (n=1), diarrhoea (n=1), myelodysplasia (thrombocytopenia and neutropenia) (n=1), behavioural disturbance (n=1), increased tremor (n=1). In six cases, reduction or withdrawal of one or two concurrent AEDs was reported. Withdrawal of stiripentol due to adverse effects occurred in three cases (23%): one due to anorexia and weight loss together with lack of efficacy, one due to behavioural disturbance and diarrhoea and one due to anorexia, unsteadiness and increased tremor. A summary of stiripentol tolerability in the audited cohort is reported in Table 2.

## 4 Discussion

In a small cohort, we show that stiripentol can be effective in adults with DS, with a reasonable tolerability profile. The responder rate in our cohort was lower than that from studies in children with DS. Not enough data on adult DS cases treated with stiripentol are available to make adequate comparisons with our findings. We note that some adults have no useful response to stiripentol.

A randomised placebo‐controlled trial conducted in children with DS^17^ showed 15 of 21 (71%) were responders (seizure‐free or experienced at least a 50% reduction in seizure frequency) on stiripentol, whereas there was only one responder of 20 (5%) on placebo (none were seizure‐free). The frequency of responders was greater on stiripentol (95% Confidence Interval 52.1–90.7) than on placebo (95% Confidence Interval 0–14.6; *P*<.001). Significantly more DS cases on stiripentol adjunctive therapy experienced drug‐related adverse events compared with patients who only had placebo add‐on (100% vs 25%; *P*=.0009). The adverse events most frequently observed on stiripentol were drowsiness and loss of appetite, sometimes causing loss of weight. The mean age of the DS cases on stiripentol was 9.4 years (interquartile range 3–16.7).

Inoue et al.^18^ performed a retrospective survey in Japan examining the effect of AEDs on clonic seizures or GTCs in DS and then started compassionate use of stiripentol as add‐on therapy in an open‐label multicenter study. They included 25 DS cases, with an older subgroup with age from 8 to 22 years (n=8). The study period lasted 6–34 months (mean 14.1). A specified protocol was used, with 2–4‐week periods of evaluation (early and late), which were compared with a 4‐week baseline period. In the older subgroup, four of eight cases (50%) had more than 50% reduction of GTCs, including one who became seizure‐free, in the early period, and five cases (63%) had more than 50% reduction of GTCs in the late period. Duration of seizures was shortened in four cases (50%) in the early period and in two cases (25%) in late period. The frequency of episodes of status epilepticus decreased in one of the eight older cases (13%). Stiripentol was discontinued in two cases (after 89 months) because seizure frequency was unchanged. The adverse effects most often observed were loss of appetite, sleep disturbance, hyperactivity or irritability, and ataxia during the early and intermediate periods. In most cases, these effects disappeared after dose modification of other AEDs/stiripentol, or in the late period.

A further prospective, open‐label, multicentre study was conducted in Japan,^19^ including 24 DS cases, already on treatment with clobazam and sodium valproate at stable doses for at least 4 weeks (bromide was also allowed as concomitant therapy). This cohort included an older subgroup aged 21–24 years (mean 23; n=4). The study consisted of a 4‐week baseline, 4‐week dose adjustment and 12‐week fixed‐dose phase. Three of the four older subjects (75%) had more than 50% reduction in frequency of clonic seizures or GTCs during the last 4 weeks of the fixed‐dose phase vs baseline, including one who became seizure‐free. After the 12‐week fixed‐dose phase, 21 of the initial 24 DS cases entered the 40‐week long‐term administration phase.^21^ None of the older cases remained responders in the long‐term (one terminated the study due to poor response without entering the long‐term phase). Adverse events were reported in all patients enrolled; the most frequent included somnolence, loss of appetite and ataxia, occurring mostly during the dose‐adjustment phase and were of mild‐to‐moderate severity.

Strzelczyk et al.^20^ retrospectively evaluated health‐care utilization over a 2‐year period in patients with DS at an outpatient clinic of a German epilepsy centre. Thirteen DS cases were included. Of these, nine (mean age 10.3, range 3–23) were switched to adjunctive treatment with stiripentol and clobazam and experienced more than 25% reduction in seizure frequency between baseline and follow‐up phases of 1 year each.

One double‐blind placebo‐controlled dose ranging study, assessing stiripentol pharmacokinetics, enrolled exclusively adult subjects (22–37 years, mean 29), but these were healthy volunteers.^22^ This study confirmed the Michaelis‐Menten pharmacokinetic profile of stiripentol that was previously described in adult healthy volunteers and people with epilepsy.^24^, ^25^ Only two adverse effects (one rhinitis and one pharyngitis) were observed, unlikely related to the treatment.

Recently, stiripentol was reported to be effective in treating super‐refractory status epilepticus, on the basis of clinical and experimental data.^26^, ^27^ Three of five adult patients who were treated with stiripentol for super‐refractory status epilepticus had resolution of status within 2–4 days after the start of stiripentol.^26^ A rodent model of established status epilepticus showed effectiveness of stiripentol when seizures had already become resistant to treatment with benzodiazepines, by potentiating GABAergic inhibition. The action of stiripentol seemed independent of the benzodiazepine binding site on the GABAA receptor.^27^, ^28^, ^29^, ^30^

The tolerability profile in our audited cohort was comparable with existing data, with anorexia, weight loss, unsteadiness and tiredness as the most common adverse effects. One case had severe haematological adverse effects with myelodysplasia (thrombocytopenia and neutropenia). However, the response to stiripentol was so dramatic (with about 12 months free of GTCs compared with GTCs occurring on a weekly basis before the introduction of stiripentol), that parents and clinicians agreed to continue the treatment, with dose adjustments and regular haematological monitoring. Lack of dose adjustments of some other concurrent AEDs at the time of the introduction of stiripentol might have affected tolerability in general, but data on concurrent AED changes were not available for all cases.

Insufficient data were available to establish a relationship with the dose of stiripentol. Due to the retrospective design of the study and the small size of the cohort, effectiveness was assessed on all seizure types and GTCs, but not on the other single seizure types. In terms of effectiveness, the responder rate (defined as more than 50% reduction in all seizure types) was 23% (3/13). More than 50% reduction in GTCs was observed in four cases (31%). It is important to note that all the reported cases had been exposed to sodium channel blockers, known to aggravate seizures and potentially precipitate status epilepticus in DS.^2^, ^6^, ^9^ This is likely related to the fact that ours was an adult cohort, with often late clinical (range 2–41 years) and genetic (range 12–43 years) diagnoses. At the time of the introduction of stiripentol, four cases were still on sodium channel blockers (i.e., oxcarbazepine, carbamazepine, lamotrigine and phenytoin). In two of these, oxcarbazepine or carbamazepine was withdrawn at the same time as stiripentol was introduced (#4 and #8). In case #3 carbamazepine was withdrawn after 5 months and in case #11 phenytoin was withdrawn 8 months after the introduction of stiripentol. Furthermore, some cases had reduction or withdrawal of other concurrent AEDs (i.e. sodium valproate, zonisamide, clobazam, topiramate) with the introduction of stiripentol (Table 2). All these concomitant AED changes might confound assessment of stiripentol effectiveness. Furthermore, eight cases (62%) had concurrent treatment with clobazam and 11 cases (85%) with sodium valproate. Stiripentol has an inhibitory effect on clobazam metabolism, mostly mediated by CYP3A4 and CYP2C19, leading to increased plasma concentrations of both clobazam and its active metabolite, norclobazam (desmethylclobazam).^17^, ^31^, ^32^ This pharmacokinetic drug interaction, along with the fact that stiripentol and clobazam/norclobazam act through separate sites on the GABAA receptor,^27^, ^28^, ^29^, ^30^ may contribute to the effectiveness as well as the adverse effects of stiripentol. Conversely, data collected in two independent multicentre randomized, double‐blind, placebo‐controlled trials in DS patients, STICLO‐France and STICLO‐Italy,^17^, ^32^ did not show any significant interaction between stiripentol and sodium valproate. Age may be another confounding factor in the assessment of the effectiveness of stiripentol. The DS adult phenotype tends to differ from the childhood pattern. Episodes of status epilepticus become rarer, atypical absences and myoclonic seizures tend to decrease, whereas focal dyscognitive seizures persist.^21^

We report seizure exacerbation in three cases (23%). The reason for this is unknown, and this outcome is not described in the stiripentol literature for adult cases and it is rarely reported in children with DS.^17^, ^18^, ^19^, ^20^, ^21^

Dravet syndrome cases are increasingly recognized in adulthood, and many children with DS survive to adulthood. Better treatment strategies are required. Stiripentol is an allosteric modulator of the GABAA receptor,^29^ proven to be effective and well‐tolerated in children with DS.^21^, ^33^ There are no data available in the literature on treatment with stiripentol of DS cases older than 24 years. Although this was a small retrospective study with incomplete information for some cases, it is the largest and oldest sample of DS adult cases treated with stiripentol and with the longest follow‐up to our knowledge. In some cases, because there were other drug changes made on clinical grounds, we cannot be certain that the entire response observed after stiripentol introduction was due solely to stiripentol: this was an audit, not a prospective clinical trial.

In view of our findings, the effectiveness demonstrated in previous studies^17^, ^21^ and its reasonable tolerability profile, we suggest stiripentol is worth considering for adults with DS and drug‐resistant epilepsy when first‐line treatments are ineffective or not tolerated, in keeping with recommendations from National Institute for Health and Care Excellence guidelines in the UK.^34^ Stiripentol use may lead to improved seizure control, with reduction of hospitalization costs and subsequent better health‐care service utilization, as previously shown.^20^ Prospective studies, including well‐designed trials in larger cohorts, would seem warranted.

## Conflict of Interests

The authors have no conflicts of interest to disclose.
